# Topical Vitamin D_3_ Derivative (Calcipotriol) Versus Intralesional Vitamin D_3_ in the Treatment of Cutaneous Warts: A Clinical Therapeutic Comparative Trial

**DOI:** 10.1155/2024/5236290

**Published:** 2024-11-05

**Authors:** Rand A. Almuhyi, Khalil I. Alhamdi, Dooha K. Alhamdi

**Affiliations:** ^1^Department of Dermatology, Alzahraa Medical College, University of Basrah, Basra, Iraq; ^2^Department of Dermatology, Basrah Medical College, University of Basrah, Basra, Iraq

## Abstract

**Background:** Cutaneous warts are epidermal proliferations caused by human papillomavirus. There are a variety of treatment options for warts with different success rates. Intralesional vitamin D_3_ injection is an innovative treatment option for warts, and several studies have examined its efficacy. To the best of our knowledge, this is the first study to compare the two modalities of vitamin D_3_ in wart treatment.

**Objective:** To evaluate and compare the efficacy of topical vitamin D_3_ derivative (calcipotriol) and intralesional vitamin D_3_ in the treatment of warts.

**Patients and Methods:** This is an open-label, therapeutic, comparative, clinical study involving 56 patients with warts. The patients were randomly divided into two equal groups (*n* = 28). Group A was treated with topical calcipotriol ointment (0.005%) twice daily for a period of 8 weeks, while Group B was treated with intralesional injection of 0.2–0.3 mL of vitamin D_3_ (300,000 I.U. per ampule) for 4 sessions (2 weeks apart). The patients were followed every 2 weeks during treatment and then 3 months after the last visit.

**Results:** The percentage of patients who showed a complete response in Group A was higher than that of Group B (95% vs. 59%). Furthermore, 9 patients out of 13 (69.2%) who showed a complete response in Group B required a period of 8 weeks, while only 2 patients out of 21 (9.5%) in Group A required the same period. In addition, side effects were more in Group B. Moreover, no recurrence was observed in Group A, while in Group B, it was seen in 2 (11%) patients.

**Conclusions:** Both topical and intralesional vitamin D_3_ are effective in the treatment of warts, with topical vitamin D_3_ having better efficacy, faster, less side effects, and less risk of recurrence.

## 1. Introduction

Cutaneous warts are common skin lesions caused by localized infection of the keratinocytes by human papillomavirus (HPV) [1]. Warts are usually asymptomatic, and spontaneous resolution occurs in about 65%–78% of the cases [2]. However, disfigurement and concerns about spreading warts to self and close contacts can cause significant embarrassment to patients; this encourages many patients to seek medical treatment rather than waiting for spontaneous clearance [3].

Wart treatment options could be categorized into ablative/cytodestructive (cryotherapy, CO2 laser, trichloroacetic acid, and electrothermal surgery) and topical treatments (podophyllotoxin, podophyllin, imiquimod, cryotherapy, and interferon) [4]. Keeping the lacunae of traditional therapies in use, immunotherapy has emerged as a novel treatment using biological substances that modulate the immune system to achieve disease control [5].

The exact mechanism behind the clearance of warts by intralesional vitamin D is not fully understood, although experimental evidence suggests that it has immunomodulatory effects by inhibiting the expression of interleukin-6, interleukin-8, tumor necrosis factor (TNF)-*α*, and TNF-*γ* mediated through the VDR-dependent pathway [6]. Topical vitamin D_3_ regulates epidermal cell proliferation and is involved in the formation of antimicrobial peptides [6]. Therefore, a synthetic vitamin D_3_ derivative (calcipotriol) was first approved by the FDA in 1993 for the treatment of psoriasis. In addition, it is a safe and effective treatment for a wide variety of diseases [7].

We conducted this study to compare the efficacy, safety profile, and recurrence rates of topical and intralesional vitamin D_3_ in the treatment of cutaneous warts. To the best of our knowledge, this is the first study of this type.

## 2. Patients and Methods

### 2.1. Participants and Study Design

**Figure 1:**
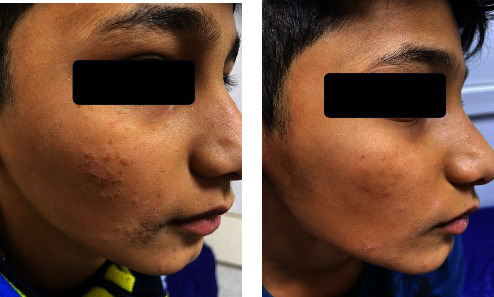
A 9-year-old male with plane warts treated with topical vitamin D_3_ showed a complete response (a) before and (b) after the treatment.

**Figure 2:**
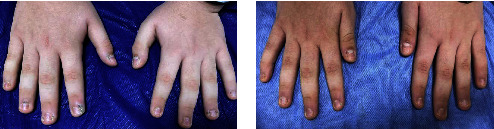
A 15-year-old male with periungual warts treated by topical vitamin D_3_ showed a complete response (a) before and (b) after the treatment.

**Figure 3:**
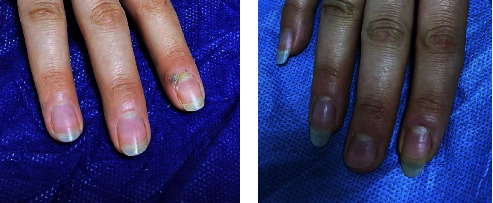
A 27-year-old female with periungual wart treated by intralesional vitamin D_3_ showed a complete response (a) before and (b) after the treatment.

**Figure 4:**
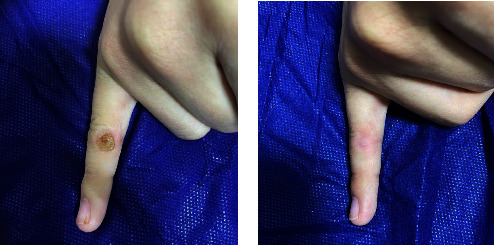
A 13-year-old male with common wart treated with topical vitamin D_3_ showed a complete response (a) before and (b) after the treatment.

**Figure 5:**
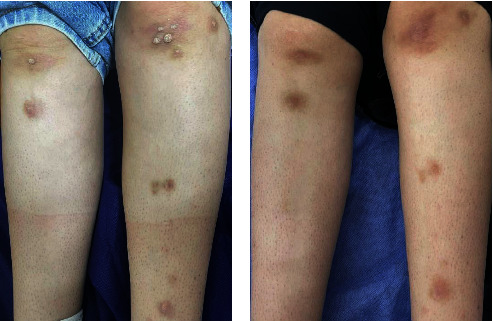
A 23-year-old female with multiple common warts treated with intralesional vitamin D_3_ showed a complete response (a) before and (b) after the treatment.

**Figure 6:**
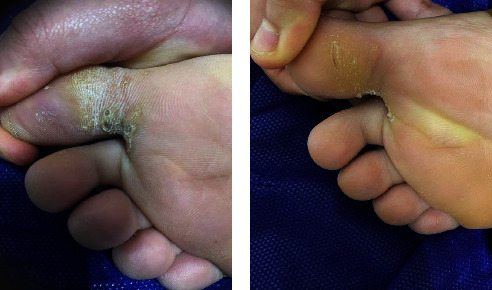
A 12-year-old female with plantar warts treated with topical vitamin D_3_ showed a complete response (a) before and (b) after the treatment.

This is an open-labeled clinical therapeutic comparative trial conducted in the dermatology outpatient clinic at Al-Sadr Teaching Hospital, Basra, Iraq, during the period from October 2022 to July 2023 after receiving approval from the University of Basrah/Alzahraa Medical College Ethics Review Committee (number MED01, dated 28/9/2022). Verbal consent was obtained from all study participants, or from their parents in the case of children, prior to enrollment and was documented using an impartial witness. For Figures 1, 2, 3, 4, 5, and 6, written informed consent for publication was also obtained.

### 2.2. Inclusion Criteria

Patients with different types and variable sizes of cutaneous warts who were diagnosed clinically and by dermoscopy, both male and female, with age older than 4 years and who had not received any treatment for viral warts in the last 2 months were included.

### 2.3. Exclusion Criteria

Patients with oral and anogenital warts, immunosuppressed patients, patients who previously received immunotherapy for their warts, pregnant or lactating women, those who have an active infection at the site of the lesion, those with a history of hypersensitivity to vitamin D_3_ or its derivatives, and patients who have diseases associated with abnormal vitamin D metabolism were excluded.

All patients were subjected to the following: the complete history included demographic data (name, age, sex, and telephone number), history of present illness, duration of the disease, previous treatments, treatment modalities, family history, drug history, and any systemic disease. Dermatological examination was performed to determine the type, site, size, and number of lesions.

### 2.4. Treatment Steps

  Group A (topical vitamin D_3_ derivative): the patients were instructed to apply 0.005% calcipotriol ointment twice a day to each wart. Follow-up was conducted every 2 weeks for 8 weeks.
  Group B (intralesional vitamin D_3_): patients in this group were treated with a slow injection of 0.2–0.3 mL of vitamin D_3_ (300,000 I.U. per ampule) at the base of each wart after applying a topical anesthetic cream (lidocaine 2.5% + prilocaine 2.5%), with a maximum amount of 1 mL used in a single session. The injections were repeated every 2 weeks interval until complete clearance or for four sessions. The patients in both groups were instructed not to use any other treatment modality during the period of this therapy.

### 2.5. Assessment and Follow-Up

All patients in both groups were examined at the first visit, clinically evaluated at subsequent visits by measuring the size of the lesions and the number of lesions and comparing them with the baseline measurements. Color photographs of each lesion were taken at the beginning and during each visit. All patients were followed up every 2 weeks for 8 weeks and after 3 months from the last visit to assess treatment response, recurrence, and occurrence of any side effects.

We adopted a clinical assessment score by recording any decrease in the size and number of warts. Patients were considered to have a complete response if all warts (100%) showed complete disappearance, a moderate response if there was more than a 50% reduction in the size and number of warts, a mild response if there was less than a 50% reduction in the size and number of lesions, and no response if there was no change (0%) in the size and number of warts [8]. Patients' satisfaction level with the results of their warts treatment was monitored on the Likert scale (Table 1). Scores were taken at the end of the study period (3 months after the last visit).

### 2.6. Calculation of the Sample Size and Randomization

Based on a previous study [10], in which complete improvement occurred in 40% of the intervention group and 5% of the control group, the number of cases necessary for conducting a two-tailed test with a significance level of *α* = 0.05 and a statistical power of 80% (*β* = 0.2) is at least 22 in each group. Patients were randomly assigned with simple randomization into two equal groups. Numbers 1–56 were placed into sealed envelopes, and participants were asked to draw one without looking at the number. Patients who drew an odd number were allocated to the topical vitamin D_3_ group, and those who drew an even number were allocated to the intralesional vitamin D_3_ group. The allocation concealment and implementation were all performed by an independent observer. The researchers and participants are all blinded to the intervention allocations.

### 2.7. Data Analysis

To analyze the data, Statistical Package for the Social Sciences (SPSS) software (Version 25.0, Armonk, NY: IBM Corp., USA) and Microsoft Excel (365) were used. Means and standard deviations (SDs) were used to describe continuous variables. Frequencies and percentages were used to describe categorical variables. Continuous variables were evaluated using *t*-tests, and categorical variables were evaluated using the Chi-square test or Fisher's exact test. *p* values < 0.05 were considered statistically significant.

## 3. Results

### 3.1. Participant Flow

**Figure 7:**
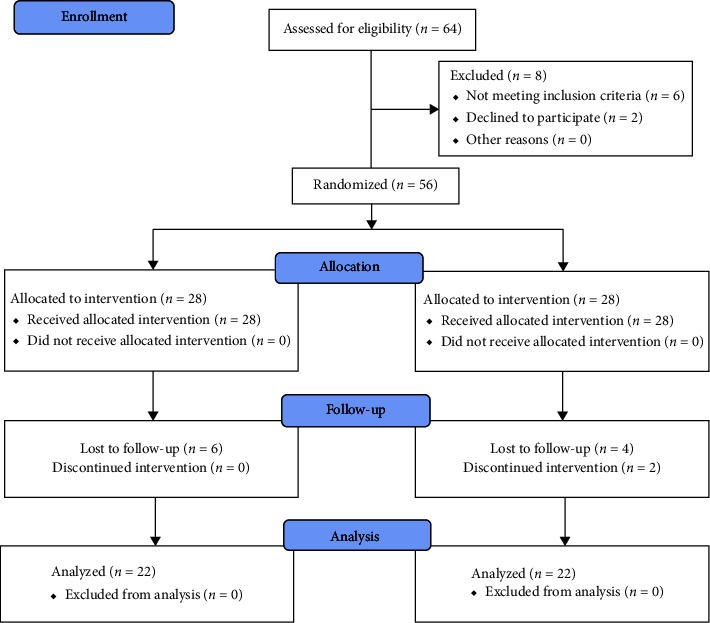
Consort flowchart.

There were 64 patients with cutaneous warts included in this trial, of which 8 were either excluded because they were unwilling to participate or failed to meet the inclusion criteria. Fifty-six patients were randomly divided into two equal groups (*n* = 28). Subsequently, six patients from the topical vitamin D_3_ group and four from the intralesional vitamin D_3_ group were lost during the follow-up due to not being present at visits, work, and other priorities, such as family commitments. Furthermore, two patients in the latter group discontinued the intervention due to experiencing pain at the time of injection, leaving 22 patients in each group for the final analysis. The flow of participants in the trial is summarized in Figure 7.

### 3.2. Demographic Characteristics of the Study Population

Patients in both groups were relatively comparable regarding the type of wart, site of wart, duration of the disease, and positive family history. However, patients in Group A were slightly older than patients in Group B with a mean of age 20 ± 9.5 and 17 ± 7.5, respectively. Also, male patients outnumbered female patients in Group A while in Group B, the reverse is true (Table 2).

### 3.3. Efficacy of the Treatment

**Figure 8:**
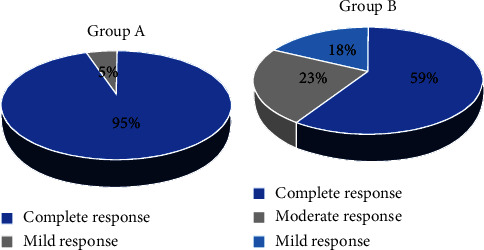
Pie-chart showing the efficacy of treatment in (a): group A (topical vitamin D group) (b): group B (intralesional vitamin D group).

Group A showed the highest efficacy, with 21 out of 22 (95%) exhibiting a complete response and only one patient (5%) showing a mild response (Figure 8(a)). In contrast, in Group B, out of 22 patients, 13 (59%) showed a complete response, 5 (23%) showed a moderate response and 4 (18%) had mild response (Figure 8(b)). The difference in the efficacy between the two groups was significant (*p* value: 0.01). Both groups had no patients with no response.

### 3.4. Complete Response on Each Visit

In addition, the percentage of patients who showed a complete response at 2 weeks and 4 weeks was significantly higher in Group A (*p* value: 0.033 and 0.01, respectively). Moreover, 69.2% of the patients who showed a complete response in Group B required a period of 8 weeks, whereas only 9.5% of the patients in Group A required the same period (Table 3).

### 3.5. Efficacy of the Treatment on Various Types of Warts

Different outcomes are observed regarding the efficacy of treatment on various types of warts in both groups (Table 4). In Group A, 100% of the patients with plane warts showed a complete response (Figure 1(a)) which is significantly higher than Group B (25%, *p* value 0.01).

Furthermore, all (100%) patients with periungual and subungual warts in Group A showed a complete response (Figures 2(a) and 2(b)) and in that of Group B, complete response was seen in 33.3% of the patients (Figures 3(a) and 3(b)). However, an equal percentage of patients with common warts (83%) (Figures 4(a) and 4(b)), (Figures 5(a) and 5(b)) and palmoplantar warts (100%) (Figures 6(a) and 6(b)) showed a complete response in both groups.

### 3.6. Side Effects in Both Groups

Pain at the time of injection was reported by almost all patients in Group B. Besides pain, the frequency of adverse events was higher in Group B (45.5%) compared with Group A (13.6%) (*p* value: 0.02). In Group B, the most common side effect was swelling, observed in 8 out of 10 patients (80%), followed by redness and itching (10% each). On the other hand, in Group A, peeling was the most common side effect, noted in 2 out of 3 patients (66.7%), and redness was observed in 1 patient (33.3%) (Table 5).

### 3.7. Patients' Satisfaction

**Figure 9:**
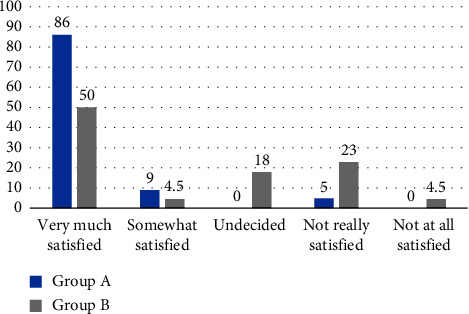
Percentage of patients' satisfaction in Group A and Group B.

A higher percentage of patients in Group A reported being very much satisfied (86% vs. 50%) compared with Group B. Notably, none of the patients in Group A expressed being not at all satisfied. In contrast, 4.5% of the patients in Group B were not at all satisfied (Figure 9). The *p* value for patient satisfaction is 0.04, indicating statistical significance.

### 3.8. Recurrence

No recurrence was observed in Group A. In contrast, recurrence was noted in 2 patients (11%) in Group B, with one patient having plane warts and the other having common warts, by the end of the follow-up period.

## 4. Discussion

The present study clearly demonstrated that both modalities were effective in the treatment of warts with topical vitamin D_3_ (Calcipotriol) being superior to intralesional vitamin D_3_ (cholecalciferol), where complete clearance of warts was achieved in 95% of the patients who were treated with topical vitamin D_3_ with an earlier response to treatment, while patients who were treated with intralesional vitamin D_3_ complete clearance was observed in 59%.

Furthermore, the duration required for complete clearance was shorter in the topical vitamin D_3_ group. At the 2-week visit, 7 out of 21 patients (33.3%) showed a complete response in the topical vitamin D_3_ group, with only 2 out of 21 patients (9.5%) requiring a period of 8 weeks. In contrast, in the intralesional vitamin D_3_ group, the majority of patients (9 out of 13, 69.2%) required 8 weeks to achieve a complete response, and only one patient (7.7%) showed a complete response after 2 weeks of treatment.

Cholecalciferol (vitamin D_3_) is an inactive vitamin D molecule that undergoes activation through two hydroxylation processes. The first occurs in the liver through the enzyme 25-hydroxylase, resulting in 25-hydroxycholecalciferol. The second occurs in the kidneys by the enzyme 1-alpha-hydroxylase, yielding the active form, 1,25-dihydroxycholecalciferol (calcitriol) [11]. In addition, keratinocytes express both enzymes and are capable of producing active calcitriol independently of renal and liver hydroxylation steps [12]. The presence of active vitamin D_3_ (calcitriol) is essential for normal keratinocyte development and function, which regulates keratinocyte proliferation, differentiation, and the formation of an intact epidermal barrier [12], although the exact mechanism underlying the therapeutic action of calcipotriol on wart is still not fully understood. However, calcipotriol is a synthetic derivative of active vitamin D_3_. Its mechanism of action is identical to its natural form, calcitriol [7], and proved to be as potent as calcitriol [13]. This probably explains the higher efficacy and shorter duration of treatment observed in patients treated with topical calcipotriol in the current study.

Vitamin D_3_ derivatives have the potential to regulate epidermal cell proliferation and differentiation and to modulate cytokine production [14]. In addition, vitamin D_3_ derivatives influence cell death, tumor invasion, and angiogenesis, making them potential agents for cancer regulation [15]. Moreover, it was recently reported that toll-like receptor (TLR) activation of human macrophages upregulated the expression of vitamin D receptor and vitamin D-1-hydroxylase genes, leading to the induction of antimicrobial peptides [14]. We surmise that these biological actions and immunomodulatory effects of calcipotriol contribute to its efficacy in treating warts, as demonstrated in the current study. Further studies are needed to confirm this.

The study also revealed fewer side effects in the topical vitamin D_3_ group, with only 3 out of 22 patients (13.6%) reporting side effects. Among these, peeling was the most common side effect observed in 2 out of 3 patients (66.7%), and redness was observed in 1 patient (33.3%). However, in the intralesional vitamin D_3_ group, pain was observed at the time of injection in almost all patients. In addition to pain, the frequency of adverse effects was higher in this group (45.5%) and the most common side effect was swelling as seen in 8 out of 10 patients (80%), followed by redness (10%) and itching (10%), all of them resolved on their own within a few days. In agreement with our study, Samta, Kumar, and Brar [6] found that pain at the time of injection and swelling were recorded in almost all patients. Furthermore, patients in the topical vitamin D_3_ group expressed greater satisfaction, 19 (86%) patients in this group were very much satisfied, and 1 patient (4.5%) was not really satisfied as a result of having a mild response. In comparison, half of the patients (11.50%) in the intralesional vitamin D_3_ group were very much satisfied, and 5 (23%) were not really satisfied. In addition, one patient was not at all satisfied despite complete clearance of the wart, attributing to the recurrence of the lesion.

In addition, no recurrence was observed in the topical vitamin D_3_ group at the end of the follow-up period, while in the intralesional vitamin D_3_ group, it was observed in 11% of the patients. In a study by Latif et al. [2] involving 41 patients, recurrence was found in 4.88% of the patients treated with intralesional vitamin D_3_.

This study demonstrated that topical vitamin D_3_ was found to be effective for all types of warts. All patients with plane warts, periungual, subangual, palmar, and plantar warts showed a complete response (100%) when treated with topical vitamin D_3_, while 83.3% of the patients with common warts had a complete response. Therefore, topical calcipotriol is a promising therapeutic option in the treatment of various types of warts, even warts that are difficult to eradicate and are known to be refractory to other treatment modalities, such as periungual and subungual warts.

A review of the literature has shown limited use of topical vitamin D_3_ in previous studies for wart treatment. For instance, Rind, Oiso, and Kawada [14] presented a case report demonstrating the successful clearance of anogenital warts in an infant using calcipotriene. Furthermore, Egawa and Ono [15] treated refractory warts in three immunocompromised patients with topical vitamin D_3._ In contrast to these studies, our study included a larger number of patients treated with topical vitamin D_3._ Furthermore, this is the first study to deal with different types of warts, differing from other studies that focused on specific types of warts when assessing the efficacy of topical vitamin D_3_.

In the current study, among patients treated with intralesional vitamin D_3_, 59% showed a complete response. In agreement with our study, Zainab et al. [16] demonstrated a comparable result (57.9%). While Kavya et al. [8] and Aktas et al. [17] found that complete clearance of warts was 78.5% and 80%, respectively.

The complete clearance achieved in the current study is slightly higher than that seen with other intralesional immunotherapy, such as *Candida* albicans (56%) [18] and the *Mycobacterium* indicus pranii vaccine (54.5%) [19].

Among the intralesional vitamin D_3_ group, 100% of the patients with palmar and plantar warts showed a complete response, while 83.3% and 33.3% of the patients with common warts and periungual warts showed a complete response, respectively. However, in a study done by Al-Sabak et al. [20], periungual warts showed significantly greater therapeutic response to intralesional vitamin D_3_ than other varieties of warts (100%). In the case of plane warts, 25% of the patients showed a complete response in the present study.

The main strength of the present study was its superiority to previous studies with respect to the comparison between the two treatment modalities. On the other hand, the study was limited by its relatively small sample size and the patients were not evaluated on the long-term effect after the end of the follow-up period, which is 3 months.

## 5. Conclusions

The vitamin D_3_ derivative (calcipotriol) is a promising option for the treatment of cutaneous warts. It has been found to be more effective in terms of efficacy, shorter duration of treatment, and no relapse with lesser side effects compared with intralesional vitamin D_3_.

## Figures and Tables

**Table 1:** Likert scale for patients' satisfaction level [9].

Patient satisfaction level	Score on Likert scale
Very much satisfied	5
Somewhat satisfied	4
Undecided	3
Not really satisfied	2
Not at all satisfied	1

**Table 2:** Demographic characteristics of study population.

Variables		Group A (topical vitamin D3) (*N* = 22) *N* (%)	Group B (intralesional vitamin D3) (*N* = 22) *N* (%)	*p* value
Age, mean ± SD		20 ± 9.5	17 ± 7.5	0.256^1^

Sex	M	12 (55%)	6 (27%)	0.065^2^
	F	10 (45%)	16 (73%)	

Duration of warts in months, mean ± SD		8 ± 7.6	10 ± 9	0.362^1^

Family history	Positive	4 (18%)	6 (27%)	0.472^2^
	Negative	18 (82%)	16 (73%)	

Type of wart	Common warts	6 (27.2%)	6 (27.2%)	
	Plane warts	6 (27.2%)	8 (36.4%)	
	Plantar and palmar warts	6 (27.2%)	4 (18.1%)	
	Periungual, subungual warts	4 (18.1%)	3 (13.6%)	
	Filiform	0 (0%)	1 (4.5%)	
Site	Face	7 (31.8%)	7 (31.8%)	
	Hands	5 (22.7%)	5 (22.7%)	
	Feet	2 (9%)	3 (13.6%)	
	Scalp	1 (4.5%)	2 (9%)	
	Palmoplantar	7 (31.8%)	4 (18.1%)	
	Legs	0 (0%)	1 (4.5%)	

*Note:p* value < 0.05 was considered statistically significant.

^1^ *t*-test was used.

^2^Chi-square test was used.

**Table 3:** Complete responses on each visit in both groups.

Visit	Group A Topical vitamin D_3_*N* = 21 (%)	Group B Intralesional vitamin D_3_ *N* = 13 (%)	*p* value
2 weeks	7 (33.3%)	1 (7.7%)	0.033
4 weeks	6 (28.6%)	0 (0%)	0.01
6 weeks	6 (28.6%)	3 (23.1%)	0.31
8 weeks	2 (9.5%)	9 (69.2%)	0.034
Total	21 (100%)	13 (100%)	

*Note:p* value < 0.05 was considered statistically significant.

**Table 4:** Efficacy of both modalities of treatment on various types of warts.

Types of warts	Response	Group A Topical vitamin D_3_ No. (%)	Group B Intralesional vitamin D_3_ No. (%)	*p* value
Common warts	Complete	5 (83.3%)	5 (83.3%)	0.9
	Moderate	0 (0%)	1 (16.7%)	
	Mild	1 (16.7%)	0 (0%)	

Plane warts	Complete	6 (100%)	2 (25%)	0.01
	Moderate	0 (0%)	1 (12.5%)	
	Mild	0 (0%)	5 (62.5%)	

Plantar and palmar warts	Complete	6 (100%)	4 (100%)	0.9
	Moderate	0 (0%)	0 (0%)	
	Mild	0 (0%)	0 (0%)	

Periungual and subungual warts	Complete	4 (100%)	1 (33.3%)	0.1
	Moderate	0 (0%)	2 (66.7%)	
	Mild	0 (0%)	0 (0%)	

Filiform warts∗	Complete	0 (0%)	1 (100%)	
	Moderate	0 (0%)	0 (0%)	
	Mild	0 (0%)	0 (0%)	

Total		22	22	

*Note:p* value < 0.05 was considered statistically significant.

^∗^There was only one patient in Group B presented with filiform warts, so there was no comparison between the two groups regarding this type of warts.

**Table 5:** Side effects' incidence in both groups.

Side effects	Group A Topical vitamin D_3_ No. (%)	Group B Intralesional vitamin D_3_ No. (%)	*p* value
Without	19 (86.4%)	12 (54.5%)	0.02
With	3 (13.6%)	10 (45.5%)	
Redness	1 (33.3%)	1 (10%)	
Itching	0 (0%)	1 (10%)	
Swelling	0 (0%)	8 (80%)	
Peeling	2 (66.7%)	0 (0%)	
All of them	0 (0%)	0 (0%)	

## Data Availability

The data used to support the findings of this study are available from the corresponding author upon reasonable request.

## References

[1] Zhu P., Qi R. Q., Yang Y (2022). Clinical Guideline for the Diagnosis and Treatment of Cutaneous Warts (2022). *Journal of Evidence-Based Medicine*.

[2] Latif I., Sultan J., Aslam A., Hassan I., Devi R. (2021). Role of Intralesional Vitamin D3 in the Treatment of Cutaneous Warts. *Journal of Cutaneous and Aesthetic Surgery*.

[3] Muhaidat J. M., Al-Qarqaz F. A., Alshiyab D. M., Alkofahi H. S., Khader Y., Ababneh M. Y. (2020). Comparison of the Efficacy and Safety of Two Cryotherapy Protocols in the Treatment of Common Viral Warts: A Prospective Observational Study. *Dermatology Research and Practice*.

[4] Mohamed E. E., Tawfik K. M., Mahmoud A. M. (2016). The Clinical Effectiveness of Intralesional Injection of 2% Zinc Sulfate Solution in the Treatment of Common Warts. *Scientific*.

[5] Mohta A., Kushwaha R. K., Agrawal A., Sharma M. K., Gautam U., Jain S. K. (2021). Evaluation of the Efficacy of Intralesional Measles, Mumps, and Rubella Vaccine With Intralesional Vitamin D3 as Immunotherapies in the Treatment of Recalcitrant Cutaneous Warts in Adult-A Randomized Placebo-Controlled Study. *Indian Dermatology Online Journal*.

[6] Samta D., Kumar S., Brar B. (2020). Intralesional Vitamin D3 for Palmoplantar Warts: A Novel Modality. *Journal of Pakistan Association of Dermatologists*.

[7] Patel R. T., Gay J. J., Fagan K. K., Eikenberg J. D. (2023). Non-Psoriatic Uses of Calcipotriol: A Concise Updated Review. *Dermatology Online Journal*.

[8] Kavya M., Shashikumar B. M., Harish M. R., Shweta B. P. (2017). Safety and Efficacy of Intralesional Vitamin D3 in Cutaneous Warts: An Open Uncontrolled Trial. *Journal of Cutaneous and Aesthetic Surgery*.

[9] Mahajan V., Singh Mehta K., Chauhan P. S., Chauhan S., Sharma V., Rawat R. (2019). Evaluation of Efficacy and Safety of Intralesional Bleomycin in the Treatment of Common Warts: Results of a Pilot Study. *Indian Journal of Dermatology Venereology and Leprology*.

[10] Kareem I. M. A., Ibrahim I. M., Mohammed S. F. F., Ahmed A. A. (2019). Effectiveness of Intralesional Vitamin D_3_ Injection in the Treatment of Common Warts: Single-Blinded Placebo-Controlled Study. *Dermatologic Therapy*.

[11] AL-Hashimi N., Abraham S. (2022). *Cholecalciferol*.

[12] Schauber J., Gallo R. L. (2008). The Vitamin D Pathway: A New Target for Control of the Skin’s Immune Response?. *Experimental Dermatology*.

[13] Wierzbicka J. M., Binek A., Ahrends T (2015). Differential Antitumor Effects of Vitamin D Analogues on Colorectal Carcinoma in Culture. *International Journal of Oncology*.

[14] Rind T., Oiso N., Kawada A. (2010). Successful Treatment of Anogenital Wart With a Topical Vitamin D (3) Derivative in an Infant. *Case Reports in Dermatology*.

[15] Egawa K., Ono T. (2004). Topical Vitamin D3 Derivatives for Recalcitrant Warts in Three Immunocompromised Patients. *British Journal of Dermatology*.

[16] Zainab Z., Malik N. A., Malik S (2021). Role of Intralesional Vitamin-D in Viral Wartse. *Journal of Ayub Medical College, Abbottabad*.

[17] Aktaş H., Ergin C., Demir B., Ekiz Ö (2016). Intralesional Vitamin D Injection May Be an Effective Treatment Option for Warts. *Journal of Cutaneous Medicine and Surgery*.

[18] Majid I., Imran S. (2013). Immunotherapy With Intralesional Candida Albicans Antigen in Resistant or Recurrent Warts: A Study. *Indian Journal of Dermatology*.

[19] Gupta S., Singh S., Chouhan K. (2014). Intralesional Immunotherapy With Killed Mycobacterium Indicus Pranii Vaccine for the Treatment of Extensive Cutaneous Warts. *Indian Journal of Dermatology Venereology and Leprology*.

[20] Al-Sabak H., Al-Hattab M., Al-Rammahi M., Al-Dhalimi M. (2023). The Efficacy of Intralesional Vitamin D3 Injection in the Treatment of Cutaneous Warts: A Clinical Therapeutic Trial Study. *Skin Research and Technology: Official Journal of International Society for Bioengineering and the Skin (ISBS)*.

